# Longitudinal Study of Metabolic Biomarkers among Conventional and Organic Farmers in Thailand

**DOI:** 10.3390/ijerph17114178

**Published:** 2020-06-11

**Authors:** Pornpimol Kongtip, Noppanun Nankongnab, Nichcha Kallayanatham, Ritthirong Pundee, Jutharak Yimsabai, Susan Woskie

**Affiliations:** 1 Department of Occupational Health and Safety, Faculty of Public Health, Mahidol University, 420/1 Rajvidhi Road, Bangkok 10400, Thailand; noppanun.nan@mahidol.ac.th (N.N.); nichcha.kal@gmail.com (N.K.); 2 Center of Excellence on Environmental Health and Toxicology, EHT, Bangkok 10400, Thailand; 3 Mahidol University, Nakhonsawan Campus, Nakhonsawan 60130, Thailand; rtg.pun@gmail.com; 4 Department of Medical Technology and Clinical Pathology, Buddhachinaraj Phitsanulok, 90 Sithamma traipidok Road, Muang, Phitsanulok 65000, Thailand; jutharak@gmail.com; 5 Department of Public Health, University of Massachusetts Lowell, One University Ave, Lowell, MA 01854-2867, USA; Susan_Woskie@uml.edu

**Keywords:** longitudinal study, cardiovascular and metabolic biomarkers, conventional farmers, organic farmers

## Abstract

The aim of this longitudinal study is to assess how pesticide use may impact metabolic biomarkers by collecting and comparing data from conventional (*n* = 13) and organic farmers (*n* = 225) every eight months for four rounds. Farmers were interviewed about family health history, food consumption behaviors, self-reported health problems, agricultural activities, and history of pesticide use. Systolic and diastolic blood pressure and body mass index (BMI) were measured. Blood samples were collected for total cholesterol, low-density lipoprotein (LDL), high-density lipoprotein (HDL), blood glucose, and triglycerides. A linear mixed model with random intercepts for subjects was used to compare the metabolic biomarkers between conventional and organic farmers and to examine the impact of the number of pesticide spray days for all four rounds after controlling for covariates. The conventional farmers reported using insecticides, herbicides, and fungicides. The marginal means for chemical farmers were significantly higher than organic farmers for total cholesterol, LDL, HDL, glucose, systolic and diastolic blood pressure, BMI, and waist circumference. Increasing the number of days of spraying either insecticides or fungicides was associated with an increase in HDL, LDL, and cholesterol levels. Increasing the number of herbicide spray days was associated with an increase in systolic and diastolic blood pressure and a decrease in BMI. These findings suggest that pesticide-using conventional farmers may be at higher risk of metabolic disease in the future.

## 1. Introduction

Pesticides are important for agricultural production; however, the widespread use of pesticides may be putting humans at risk for adverse health effects [1]. In Thailand, 131,308 tons of pesticides were imported in 2019, including 88,846 tons of herbicides, 16,897 tons of insecticides, 19,334 tons of fungicides, and 6231 tons of other pesticides [2]. In Thailand, acute poisoning remains a risk factor for pesticide use. We had 4001 pesticide-poisoning cases and 520 deaths during the period of October 2018 to June 2019 [2,3]. However, little work has been done to investigate the potential for chronic health effects from pesticide use by farmers in Thailand.

The organic farming sector in Thailand is in early development. The main organic agricultural products are rice, vegetables, and fruits; the area under organic farming is slowly increasing from 0.2% of the total agricultural area in Thailand in 2011 [4]. Farmers adopt organic farming based on knowledge of organic farming systems, market costs and dynamics, and the environmental benefits to the farm [5]. In addition, the health benefits to the farmers of reduced pesticide exposure and to consumers of reduced pesticide contamination in the food supply also play a role in the conversion of farms to organic agriculture [6]. Previously, we have reported results from a cross-sectional study of conventional and organic farmers, where we found that conventional pesticide-using farmers had a significantly higher risk of abnormal metabolic and cardiovascular health outcomes and biomarkers, compared to organic farmers [7]. This study follows that cohort for three years, with four rounds of data collection, to assess whether the cross-sectional finding results remain consistent over time.

## 2. Materials and Methods

### 2.1. Study Population and Data Collection

To collect different groups of conventional as well as organic farmers, we recruited farmers from three provinces in Thailand. For conventional farmers using pesticides, we recruited rice and vegetable farmers in two subdistricts, Bueng Phra Subdistrict and Wat Phrik Subdistrict, Muang in Phitsanulok province, located in the lower north of Thailand. Sugarcane and rice farmers in Khao Thong Subdistrict, Phayuha Khiri District in Nakorn Sawan province, are located in the upper central area in Thailand. For conventional farmers, one farmer who sprayed pesticides was selected in each household. Organic farmers were recruited from five subdistricts: Sang Ming subdistrict, Loeng Nok Tha district, Kammaet and Na So subdistricts, Kut Chum district, and Bak Ruea subdistrict, Maha Chana Chai district in the Yasothorn province, in northeast Thailand. They had to use certified organic farming practices for all of their crops, including rice, vegetables, and fruits. One farmer was selected from each household.

The studied subjects were male or female farmers over 18 years old who were free of a current diagnosis of diabetes, high blood pressure, and thyroid or heart disease. Health-promoting hospitals/primary care units (HPH/PCUs) in the areas, community leaders, public health volunteers, and the hired site officer worked together to help recruit farmers. The site officer visited each home during the recruitment to conduct interviews during the two months before physical health examinations in each round. Physical examinations and blood collection occurred at the local HPH/PCU. The subjects received a small remuneration for participation in this study, and a study doctor explained the results of their individual health checkup. At recruitment, we enrolled 243 conventional farmers and 235 organic farmers.

The questionnaires comprised screening questions, demographics, health behaviors, family health history, food consumption behaviors, home location and home pesticide use, self-reported health problems, agricultural activities, and history of pesticide use. The Department of Mental Health stress questionnaire (Department of Mental Health, Thai Ministry of Public Health) [8] was used and covered sleeping problems, reduced concentration, irritability, boredom, and lack of interest in meeting people, with a scale of 0 for no problem to 3 as always having problems. In the morning (7–9 a.m.), during the physical exam, data on weight, height, waist circumference, body composition (Tanita model DC-360, Amsterdam, Netherland), blood pressure (taken twice, 10 min apart and averaged), and blood samples were collected. The farmers were told to abstain from food for at least 8 h before the blood collection. Sera extracted from blood samples in nonheparinized vacutainer tubes were stored at −20 °C until analysis. Serum glucose, triglycerides (TGs), total cholesterol (TC), high-density lipoprotein (HDL), and low-density lipoprotein (LDL) were measured by an AU5800 apparatus (Beckmann Coulter, Atlanta, GA, USA) at Buddhachinaraj Hospital, the regional medical center in the Phitsanulok province, using standard clinical laboratory methods. The blood sample collection and physical health examination were collected over four rounds, 8 months apart. The first round was November to December 2016, 8 months after recruitment. The second round was in July 2017, the third round in April to May 2018, and the fourth round in January to February 2019.

All subjects gave their informed consent for inclusion before they participated in the study. The study was conducted in accordance with the Declaration of Helsinki, and the protocol was approved by the Ethics Committee of Human Research, Faculty of Public Health, Mahidol University (MUPH 2015-146).

### 2.2. Statistical Analyses

All statistical analyses were done by SPSS for Windows, version 23 (IBM Thailand Co., Ltd., Bangkok, Thailand). When comparing conventional and organic farmers, contingency tables, chi-square, Fisher’s exact test, and independent *t*-tests were employed. To account for the repeated measures on each subject, a linear mixed model with random intercepts for subjects was used to estimate the marginal means for each of the metabolic biomarkers for the organic and conventional farmers, after controlling for baseline age, gender, smoking, alcohol and pesticide use at home [7]. We also used a linear mixed model with random intercepts for subjects to look at the change in biomarker levels with an increasing number of self-reported days of spraying in the previous 8 months, for each type of pesticide, after controlling for the same covariates and the round of data collection.

## 3. Results

When the first physical health checkups were conducted, 8 months after recruitment, the cohort had declined from 243 to 213 conventional farmers and from 235 to 225 organic farmers, with response rates of 87.7% and 95.7%, respectively. In the second, third, and fourth rounds, collected about 8 months apart, the loss of follow-up was 4.57%, 4.78%, and 4.77%, respectively.

### 3.1. Characteristics of Subjects and Metabolic Biomarkers

The average age and education of organic farmers were significantly higher than those of conventional farmers (Table 1).

The conventional farmers comprised a higher percentage of male farmers, while the organic farmers had a similar male to female ratio. The organic farmers held second jobs more frequently than the conventional farmers and worked more hours per week in their agricultural work as well as their second job. The conventional farmers were significantly more likely to be smokers, drinkers, and use insecticides at home in the past year.

### 3.2. Metabolic Biomarkers for Organic versus Conventional Farmers

The marginal means estimated from repeated measures linear mixed models showed that conventional farmers had significantly higher total cholesterol, LDL, HDL, blood glucose, triglycerides, systolic and diastolic blood pressure, BMI and waist circumference than organic farmers, accounting for all 4 rounds of data collection (Table 2).

### 3.3. Pesticide Spraying Days of Conventional Farmers

Organic farmers did not report spraying chemical pesticides. The conventional farmers self-reported the number of days of spraying by crop season, which was used to calculate the number of days of spraying insecticides, herbicides, and fungicides in the previous 8 months before the biomarker collection. The pesticide-spraying days (median and Interquartile Range, IQR) are presented in Table 3 for each round.

The mean number of spray days was highest for Round 1 for insecticides and fungicides, and highest in Round 2 for insecticides. The lowest mean number of spray days was in Round 4 for insecticides and fungicides. It should be noted that the median spray days for fungicides for all rounds was zero so that the mean was highly influenced by a few high values, while a median of 4 spray days was reported for insecticides for all rounds and for all but Round 2 (8) for herbicides.

### 3.4. Change of Metabolic Biomarkers per 10 Days of Pesticide Sprayed

The change in metabolic biomarkers per an increase in 10 days of pesticide spraying is presented in Table 4. An increase in days of insecticide spraying was significantly associated with increased HDL and LDL, and a borderline significant increase in total cholesterol. An increase in herbicide spraying was significantly associated with an increase in HDL and systolic and diastolic blood pressure, but a decrease in BMI. An increase in fungicide-spraying days was significantly associated with an increase in total cholesterol, HDL, and LDL.

## 4. Discussion

The characteristics of the farmers enrolled in this study showed that conventional farmers were significantly more likely to be smokers and drinkers and use pesticides at home, and have lower education compared to organic farmers. Chouichom (2010) [5] reported that longer-term farm experience (higher age) and higher education were supporting factors in the choice to transfer to an organic farming system [9]. Training in organic farming emphasizes a better quality of life and organic farmers tend to have lower rates of smoking and drinking. In addition, the conventional farmers in this study were more likely to be male, whereas the organic farmers were equally divided between males and females. In Thailand, alcohol consumption and smoking are more likely to occur with males than females [10,11].

In our previous cross-sectional study, we found that conventional farmers who currently use pesticides had a significantly higher risk of abnormal metabolic and cardiovascular health biomarkers, including clinically abnormal levels of triglycerides, total cholesterol, LDL, waist circumference, % body fat, and BMI, as compared to organic farmers, after controlling for a variety of demographic and behavioral risk factors [7]. In this longitudinal study, the conventional and organic farmers were followed-up for three years, with four rounds of physical health outcomes and questionnaires. We compared actual levels of the biomarkers between organic and chemical farmers (not the proportion of clinically abnormal results). However, we found complementary results for BMI, cholesterol, HDL, and LDL, where conventional farmers had statistically significantly higher levels of these measures. Unlike our previous study, we did not find triglycerides or % body fat were significantly higher in conventional farmers.

With regard to organic versus conventional farmers, our findings are similar for lipids and glucose but different for triglycerides from those of animal studies, where after exposure to malathion, rats were found to have significantly higher serum total cholesterol, and other liver enzymes and significantly lower triglycerides and very low-density lipoprotein levels compared to controls [12]. In this longitudinal study, there were no significant differences in triglyceride levels between organic and conventional farmers, although lipids were significantly higher. Likewise, in rats, chlorpyrifos has been observed to induce significant increase in total cholesterol, but a significant decrease in triglyceride levels [13]. However, rats exposed to monocrotophos had significantly decreased plasma triglyceride levels in an earlier phase (50–60% of control), followed by hypertriglyceridemia in a later phase (15–30% above control) [14].

We did find that blood glucose and systolic and diastolic blood pressure were significantly higher in conventional farmers, and this aligns with the findings of animal studies that show hyperglycemia following exposure to organophosphates and other pesticides [13,15,16]. Exposure to organophosphates, organochlorines, and carbamates during the first trimester of pregnancy was found to increase the risk of gestational diabetes mellitus (GDM) [17]. In rats exposed to acephate orally, blood glucose increased in 2 h, with a significant increase in liver glycogen content 6 h after exposure [18]. Repeated exposure to very high doses of dimethoate and diazinon were also shown to cause a significant increase in blood glucose [19,20]. It was reported that malathion induces hyperglycemia mainly through altering glucose metabolism in the liver and skeletal muscles. Malathion also activates islets muscarinic receptors, resulting in hyperinsulinemia, overproduction of glutamate/glutathione by glutamate dehydrogenase (GDH), and overproduction of glucose via increased glycogenolysis in response to malathion-induced oxidative stress [21]. In humans, exposure to insecticides is linked to an increased risk of obesity and type 2 diabetes [22,23,24]. Pesticides may induce metabolic disorders, such as diabetes and obesity, by disturbing energy absorption in the intestine, energy storage in the liver, and by affecting fat tissue, skeletal muscle, the pancreas, and immune cells [25].

The conventional farmers in this study were sugarcane, rice, and vegetable farmers. Most reported spraying several types of pesticides, including herbicides, insecticides, and fungicides. In the first 8 months, the herbicides reported included glyphosate, paraquat, 2,4-D sodium salt, diuron, amethrin, acetochlor, and propanil. The insecticides used included chlorpyrifos, methyl parathion, carbofuran, carbosulfan, cypermethrin, abamectin, and profenofos. The fungicides used included azoxystrobin, difenoconazole, propiconazole, propineb, carbendazim, isoprothiolane, and mancozeb [7]. The median of insecticide-spraying days was the same for 4 rounds, but due to the skewed distribution of spray days, the average number of spraying days declined from Round 1 to Round 4. The number of days herbicides were sprayed was highest in the second round. Compared to the other pesticides, the use of fungicides was the lowest, with the median days sprayed equal to zero for all rounds, although due to the skewed distribution of spray days, the average number of spray days was lowest in Round 4. There are many reports of problems with pesticide use in developing countries related to overuse and misuse, lack of training in hazards and proper pesticide use, inappropriate pesticides being used due to manufacturer-to-retailer subsidies, and inadequate regulation and enforcement [26,27].

More frequent application of insecticides and fungicides resulted in significantly higher total cholesterol, HDL, and LDL for the conventional farmers compared to the organic farmers (borderline with total cholesterol and insecticides). Aminov et al. 2013 [28] reported that in residents of Anniston, Alabama, organochlorine pesticide concentrations were associated with an increase in total serum lipids, and total cholesterol and triglycerides. In developing countries, higher socioeconomic status has been associated with higher levels of serum lipids [29,30]. However, in the current study, the education level of organic farmers was significantly higher than those of conventional farmers, but the organic farmers had significantly lower serum lipids, including total cholesterol, LDL, HDL, and BMI than the conventional farmers. Self-reported income adequacy was not significantly different between the two groups. For nutrition, the organic farmers reported consuming significantly more sweet fruits, snacks, and desserts than the conventional farmers, but the consumption of other types of food, such as meat with high fat and deep-fried food, was not significantly different between the two groups [7].

Du et al. (2013) [31] concluded that organophosphates at low doses cause liver dysfunction, which affects the tricarboxylic acid cycle and the lipid metabolism of rats. The liver is the primary detoxification organ of xenobiotic pesticides in the body, with p450-mediated enzymatic catalysis eventually inhibited due to pesticide-induced liver damage and cell death [25]. Chlorpyrifos and deltamethrin cause liver damage [32]. Consequently, the pesticides and exogenous toxins travel from the liver to other fat-rich tissues, such as adipose tissue, causing more severe negative effects on energy metabolism and other body functions. Exposure to β-HCH reduced β-oxidation and increased fatty acid synthesis in the livers of mice [33]. Some pesticides can accumulate in the adipose tissue, causing obesogenic effects. Exposure to chlorpyrifos [34] and imidacloprid [35,36] increased the epididymal fat weight in mice fed a high-fat diet. Exposure to permethrin [37], diazinon [38], deltamethrin [39], chlorantraniliprole [40], and fipronil [41] were found to induce lipid accumulation by regulating the AMP-activated protein kinase (AMPK) pathway in mice. Male zebrafish exposed to the fungicide tebuconazole for 7 days showed increases in glucose, cholesterol, triglycerides, and lactate levels over those in the control fish [42]. Pesticide-induced changes in blood lipid levels are associated with several metabolic disorders, which, in turn, lead to fibrosis, atherosclerosis, and cardiac disease [43]. In humans with chronic pesticide use, serum cholesterol, triglycerides, and LDL levels were increased significantly among those with decreased cholinesterase levels compared to persons with normal cholinesterase levels [44].

We also found that the increasing use of herbicides resulted in significant increases in HDL and systolic and diastolic blood pressure and decreased BMI. Among US Vietnam veterans tasked with aerial spraying of herbicides during the war, herbicide-spray history was significantly associated with self-reported physician-diagnosed hypertension [45]. Similarly, Yi et al. (2013) [46] found a statistically significant association of hypertension and self-reported Agent Orange exposure (2,4,5-T and 2,4-D herbicides contaminated with dioxin). It is unclear whether exposure was an initiating or synergistic factor in the development of hypertension. Dioxins are believed to initiate blood pressure changes through the endothelial aryl hydrocarbon receptor (AhR), but this mechanism has been proved only in an animal model [47]. Samsuddin et al. (2015) [48] reported that chronic mixed pesticide exposure among workers involved in mosquito control was possibly associated with depression of diazoxonase and an increase in oxidized low-density lipoproteino (x-LDL), brachial and aortic diastolic BP, systolic BP, and heart rate.

Pesticides may be acting as endocrine-disrupting chemicals (EDCs), which are known to interfere with hormonal action and impair hormone functions in the regulation of development and the maintenance of homeostasis and physiological processes, including adipogenesis and energy balance [49,50]. We report that conventional farmers had significantly higher BMI and waist circumference than organic farmers but did not find a significant association between the number of spray days and BMI, except for with herbicides, where the association was negative (lower BMI with increasing use). Perinatal exposure to EDCs and other environmental pollutants has been proposed as a contributing factor to childhood overweight and obesity [51,52]. Lind et al. (2013) [53] demonstrated that long term weight gain was observed in older subjects with higher serum levels of summed organochlorine pesticides and higher levels of low-chlorinated PCBs relative to those with lower serum levels of these compounds. The development of metabolic syndrome was associated with exposure to organochlorine pesticides in Anniston, Alabama, residents without diabetes [54,55]. Berg et al. (2019) [56] reported that occupational exposure to pesticides (organophosphate and carbamates insecticides) may play a role in the development of cardiovascular diseases (CVDs). They report that people with other cardiovascular confounders such as obesity, hypertension, and stroke were more likely to have exacerbations of their health conditions when exposed to agrochemicals over a period of time.

Limitations of our study include potential misclassification of pesticide use, which was retrospectively collected from farmers by questionnaire (covering the previous growing seasons). Repeated measures of pesticide biomarker levels could improve the exposure assessment if taken frequently throughout the study period. This longitudinal study covered four rounds of data collection, eight months apart. Some biomarkers like body fat percent may change more slowly, and the development of metabolic syndrome or other outcomes of metabolic disorders like stroke, diabetes, and cardiovascular disease are likely to require a longer follow-up period. Although we controlled for some factors like age, gender, smoking, and alcohol use in our analyses, there may be other unidentified confounders that could explain the differences in metabolic biomarkers between organic and conventional farmers [7].

## 5. Conclusions

In this longitudinal study, conventional farmers using pesticides had significantly higher total cholesterol, LDL, HDL, blood glucose, systolic and diastolic blood pressures, BMI, and waist circumference than those of organic farmers. Increasing the number of days spraying pesticides (insecticides, herbicides, or fungicides) in the previous eight months was associated with increases in lipid levels. Increasing days of herbicide-spraying was associated with changes in blood pressure and decreases in BMI.

## Figures and Tables

**Table 1:** Characteristic and risk factors of conventional farmers (*n* = 213) and organic farmers (*n* = 225).

Variables		Conventional Farmers *n* (%)	Organic Farmers *n* (%)	*p*-Value
Age				
	Min–max	18–69	28–79	
	Mean (SD)	50.22 (11.1)	53.20 (10.3)	0.005 §
Sex				
	Male	158 (74.2)	115 (51.1)	<0.001 †
	Female	55 (25.8)	110 (48.9)	
Educational level				
	Below elementary	14 (6.6)	4 (1.8)	0.035 †
	Elementary	122 (57.3)	125 (55.6)	
	High school	72 (33.8)	85 (37.8)	
	Bachelor or higher	5 (2.3)	11 (4.9)	
Marital status				
	Single	21 (10.1)	13 (6)	0.032 †
	Married	179 (86.1)	185 (84.9)	
	Widowed/divorced	8 (3.8)	20 (9.2)	
Agricultural work time (h/week)				
	Mean (SD)	26.9 (13.8)	28.8 (17.2)	0.011 §
Have Second Job				
	Yes	49 (23)	128 (56.9)	<0.001 †
	No	164 (77)	97 (43.1)	
Second job work time (h/week)				
	Mean (SD)	24.9 (13.6)	26.6 (17.8)	0.048 §
Alcohol intake				
	Current drinker	136 (63.8)	91 (41)	<0.001 †
	Non drinker	77 (36.2)	131 (59)	
Expense adequacy				
	Enough for saving	39 (18.3)	50 (22.2)	0.618 †
	Just enough	100 (46.9)	94 (41.8)	
	In debt	73 (34.3)	79 (35.1)	
Smoking				
	Current smoker	59 (26.9)	36 (16.1)	0.006 †
	Non smoker	155 (73.1)	188 (83.9)	
Any stress symptom in past 2–4 weeks				
	Yes	113 (53.3)	100 (45.0)	0.085 †
	No	99 (46.7)	122 (55.0)	
Insecticide use in home in the past year				
	Yes	191 (89.7)	33 (14.7)	<0.001 †
	No	22 (10.3)	191 (85.3)	

† = chi-square, § = independence *t*-test.

**Table 2:** Estimated marginal means for 4 rounds of measurement of organic and chemical farmers.

Parameter	Conventional Farmer Mean (95%CI)	Organic Farmer Mean (95%CI)	*p*-Value from Model F-Test
Total Cholesterol (mg/dL)	217.9 (211.2–224.6)	193.0 (186.6–199.3)	<0.001
LDL (mg/dL)	132.1 (126.9–137.3)	117.8 (112.7–122.2)	<0.001
HDL (mg/dL)	56.5 (54.6–58.3)	45.8 (44.1–47.6)	<0.001
Blood glucose (mg/dL)	109.9 (106.7–113.0)	104.1 (101.1–107.1)	0.009
Triglyceride (mg/dL)	157.2 (142.3–172.1)	147.6 (133.5–161.7)	0.356
Systolic BP (mmHg)	134.5 (131.9–136.6)	129.3 (127.1–131.5)	0.002
Diastolic BP (mmHg)	80.7 (79.3–82.1)	76.8 (75.4–78.1)	<0.001
BMI (kg/m^2^)	24.3 (23.7–24.9)	23.3 (22.8–23.8)	0.010
Body fat (%)	28.0 (27.0–29.1)	27.5 (26.4–28.5)	0.469
Waist circumference (cm)	83.6 (82.1–85.1)	80.7 (79.2–82.1)	0.004

Marginal mean estimated from repeated measures linear mixed model with random intercepts for subjects and fixed effects for organic vs. conventional farmer, along with covariates for age at baseline, gender, smoking use, alcohol use, pesticide use at home.

**Table 3:** Self-reported pesticide exposure (days of spraying) for each round (8 months) among conventional farmers.

Days of Spraying Pesticide	Round 1 Mean (IQR) *n* = 213	Round 2 Mean (IQR) *n* = 199	Round 3 Mean (IQR) *n* = 185	Round 4 Mean (IQR) *n* = 178
Insecticide	14 (4–24)	11 (2–24)	11 (2–8)	7 (0–4)
Herbicide	7 (4–8)	9 (4–14)	7 (4–12)	8 (2–12)
Fungicide	9 (0–8)	7 (0–8)	8 (0–8)	3 (0–2)

IQR: Interquartile Range.

**Table 4:** Change in metabolic biomarkers per 10 days of pesticide sprayed (β × 10) in the previous 8 months from a linear mixed model with random intercepts for subjects and fixed effects for days of a type of pesticide sprayed (insecticide or herbicide or fungicide), along with covariates including baseline age, gender, smoking, alcohol and pesticide use at home, and testing round.

Metabolic Biomarker	Insecticide Spray Days	*p*-Value	Herbicide Spray Days	*p*-Value	Fungicide Spray Days	*p*-Value
Total cholesterol (mg/dL)	1.78 (−0.01, 3.56)	0.052	1.76 (−1.2, 4.71)	0.243	2.15 (0.07, 4.24)	0.043
HDL (mg/dL)	0.96 (0.42, 1.49)	<0.001	1.42 (0.53, 2.32)	0.002	1.25 (0.63, 1.87)	<0.001
LDL (mg/dL)	1.56 (0.16, 2.96)	0.028	1.11 (−1.20, 0.34)	0.345	1.75 (0.12, 3.38)	0.035
Glucose (mg/dL)	0.29 (−0.51, 1.08)	0.482	−0.60 (−1.88, 0.68)	0.358	0.13 (−0.79, 1.05)	0.782
Triglyceride (mg/dL)	−2.80 (−7.47, 1.87)	0.239	−3.22 (−10.92, 4.49)	0.413	−2.57 (−8.02, 2.87)	0.354
Systolic Blood Pressure (mmHg)	0.19 (−0.39, 0.78)	0.519	1.14 (0.19, 2.09)	0.018	0.16 (−0.52, 0.83)	0.649
Diastolic Blood Pressure (mmHg)	−0.01 (−0.35, 0.32)	0.937	0.58 (0.04, 1.13)	0.037	0.16 (−0.23, 0.55)	0.412
BMI (kg/m^2^)	−0.03 (−0.10, 0.04)	0.453	−0.14 (−0.26, −0.03)	0.015	−0.03 (−0.12, 0.05)	0.444
Body fat (%)	0.11 (−0.17, 0.4)	0.449	−0.25 (−0.72, 0.22)	0.300	0.10 (−0.24, 0.43)	0.573
Waist Circumference (cm)	0.20 (−0.10, 0.49)	0.171	−0.02 (−0.48, 0.45)	0.946	0.14 (−0.19, 0.48)	0.402
